# Supplementary material for: Comparison of Whole Blood Cryopreservation Methods for Extensive Flow Cytometry Immunophenotyping
Source: Cells. 2022 May 2;11(9):1527. doi: 10.3390/cells11091527 (PMC9103885; doi:10.3390/cells11091527)
Supplement: Supplementary file 1 [file cells-11-01527-s001.zip › Supplementary Figure S1.pdf]

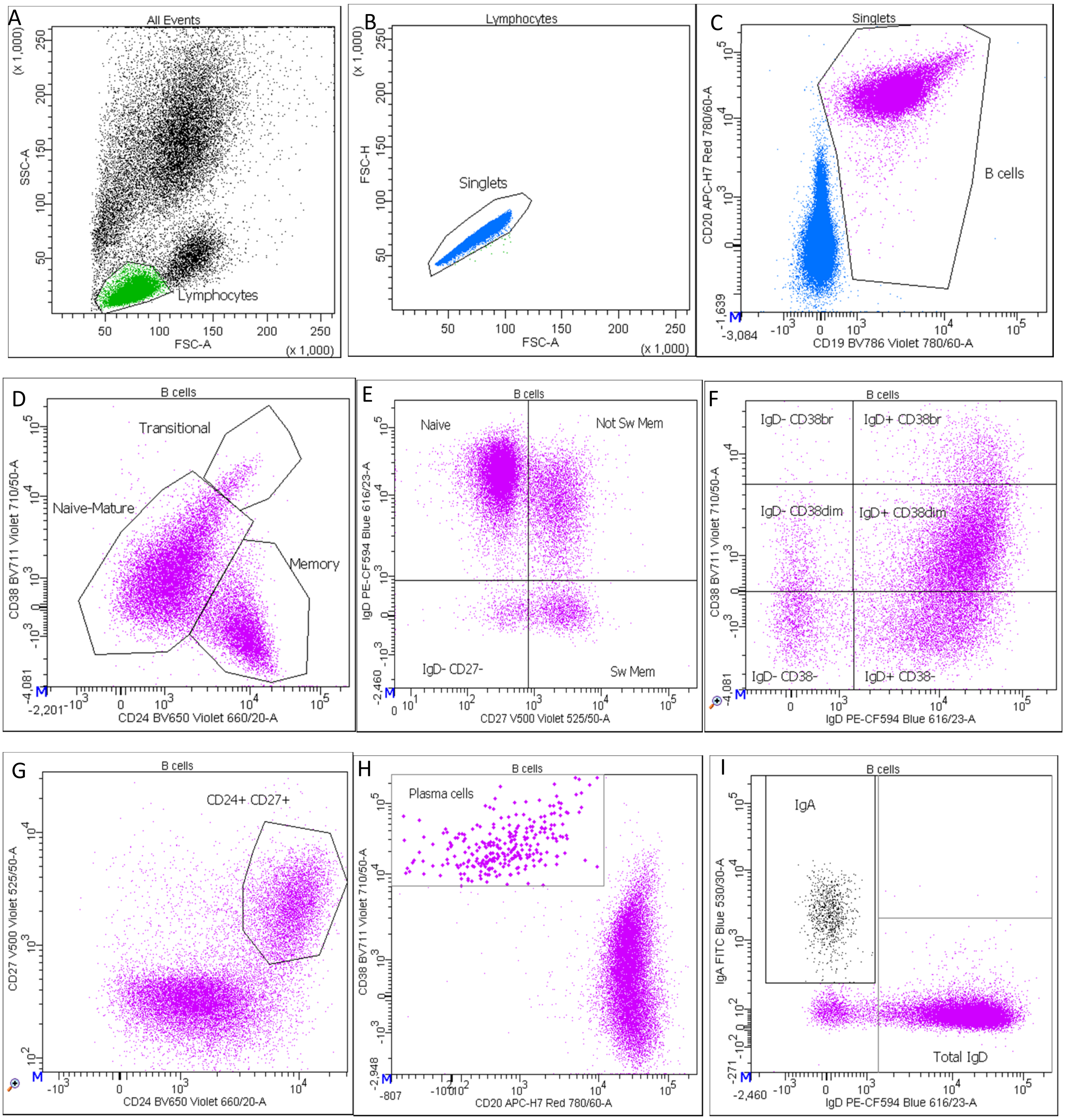

Supplementary Figure S1. B cell gating strategy representative of a fresh processed sample. (A-B) Forward scatter (FSC) vs Side scatter (SSC) were used to morphologically identify lymphocytes (green). (C) B cells (purple) were identified as CD19 positive lymphocytes, then classified using different approaches: (D) CD24 vs CD38 discriminating transitional (CD24+ CD38hi), memory (CD24+ CD38−/dim) and naïve-mature (CD24− CD38−/dim) subsets; (E) CD27 vs IgD identifying switched memory (CD27+ IgD−), un-switched memory (CD27+ IgD+), naïve (CD27− IgD+) and CD27− IgD− B cells; (F) IgD vs CD38 distinguishing Bm1 (IgD+ CD38−), Bm2 (IgD+ CD38dim), Bm2′ (IgD+ CD38br), Bm3-Bm4 (IgD- CD38br), early Bm5 (IgD- CD38dim), and late Bm5 (IgD- CD38−) memory cells; (G) CD24 vs CD27 identifying CD24+ CD27+ memory cells; (H) CD20 vs CD38 discriminating plasma blasts/plasma cells as CD20− CD38hi; (I) IgA vs IgD identifying IgA+ B cells and IgD+ B cells.
