# Supplementary material for: Comparison of Whole Blood Cryopreservation Methods for Extensive Flow Cytometry Immunophenotyping
Source: Cells. 2022 May 2;11(9):1527. doi: 10.3390/cells11091527 (PMC9103885; doi:10.3390/cells11091527)
Supplement: Supplementary file 1 [file cells-11-01527-s001.zip › Supplementary Figure S2.pdf]

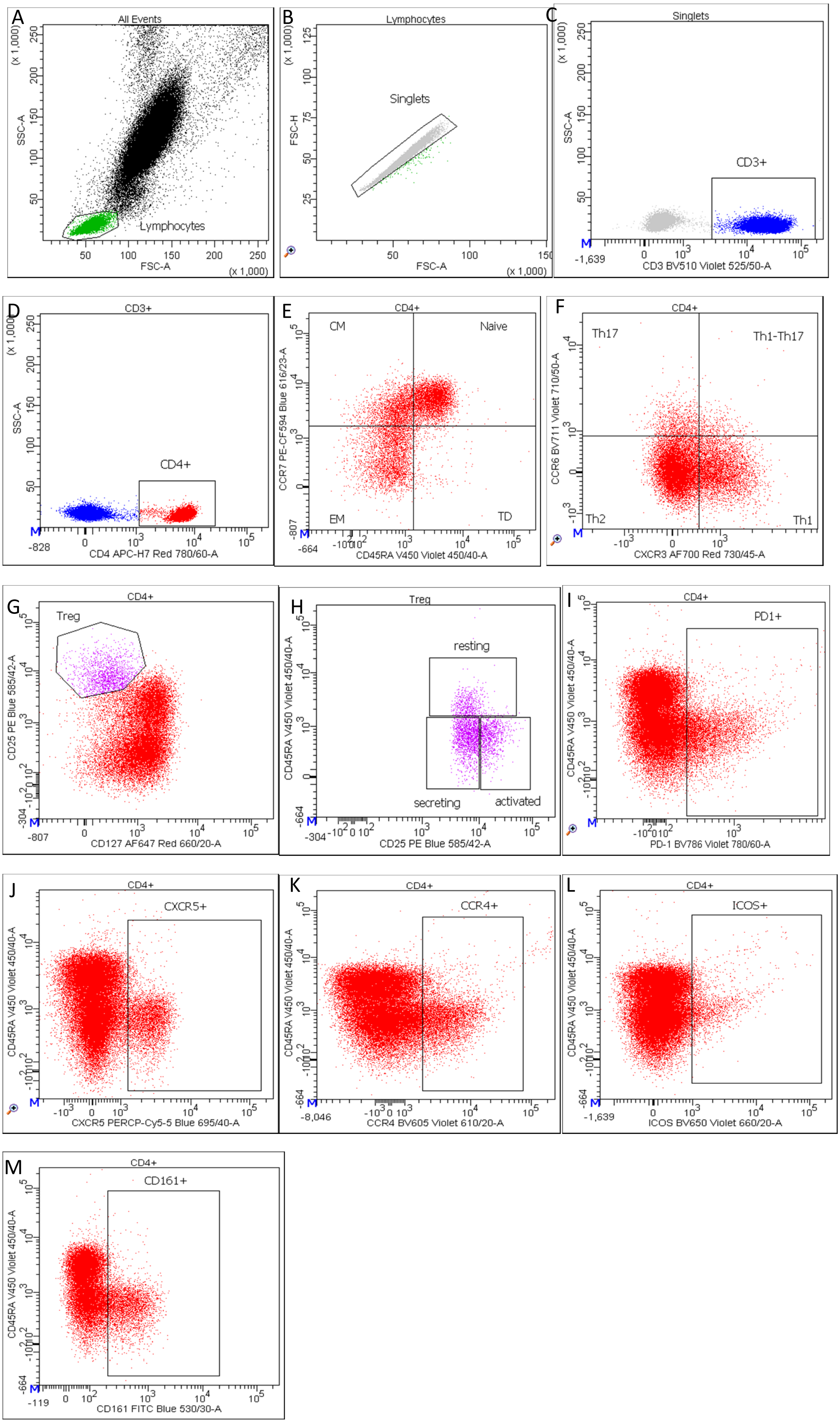
A
All Events
Lymphocytes
SSC-A
FSC-A
B
Lymphocytes
Singlets
FSC-H
FSC-A
C
Singlets
CD3+
SSC-A
CD3 BV510 Violet 525/50-A
D
CD3+
CD4+
SSC-A
CD4 APC-H7 Red 780/60-A
E
CD4+
CM
Naive
EM
TD
CCR7 PE-CF594 Blue 616/23-A
CD45RA V450 Violet 450/40-A
F
CD4+
Th17
Th1-Th17
Th2
Th1
CCR6 BV711 Violet 710/50-A
CXCR3 AF700 Red 730/45-A
G
CD4+
Treg
CD25 PE Blue 585/42-A
CD127 AF647 Red 660/20-A
H
Treg
resting
secreting
activated
CD45RA V450 Violet 450/40-A
CD25 PE Blue 585/42-A
I
CD4+
PD1+
CD45RA V450 Violet 450/40-A
PD-1 BV786 Violet 780/60-A
J
CD4+
CXCR5+
CD45RA V450 Violet 450/40-A
CXCR5 PERCP-Cy5-5 Blue 695/40-A
K
CD4+
CCR4+
CD45RA V450 Violet 450/40-A
CCR4 BV605 Violet 610/20-A
L
CD4+
ICOS+
CD45RA V450 Violet 450/40-A
ICOS BV650 Violet 660/20-A
M
CD4+
CD161+
CD45RA V450 Violet 450/40-A
CD161 FITC Blue 530/30-A

Supplementary Figure S2. CD4+ T cell gating strategy representative of a fresh processed sample. (A-B) Lymphocytes (green) were identified by the morphological parameters; (C) CD3+lymphocytes (blue) corresponding to T cells; (D) CD4+ T cells (red). (E) The maturation stages of CD4+ T cells were assessed basing on CCR7 and CD45RA expression into CM (CD45RA− CCR7+), naïve (CD45RA+ CCR7+), TD (CD45RA+ CCR7−) and EM (CD45RA− CCR7−). (F) The expression of CCR6 and CXCR3 on CD4+ T cells distinguished Th17 (CXCR3− CCR6+), Th1-17 (CXCR3+ CCR6+), Th1(CXCR3+ CCR6−) and Th2 (CXCR3− CCR6−). (G) Tregs (purple) were identified as CD4+ T cells expressing high levels of CD25 and low levels of CD127 antigens and (H) further subdivided in resting (CD45RA+ CD25+), activated (CD45RA− CD25++) and secreting (CD45RA− CD25+). CD4+ T cells expressing (I) PD1, (J) CXCR5, (K) CCR4, (L) ICOS and (M) CD161 antigens.
