# Supplementary material for: Comparison of Whole Blood Cryopreservation Methods for Extensive Flow Cytometry Immunophenotyping
Source: Cells. 2022 May 2;11(9):1527. doi: 10.3390/cells11091527 (PMC9103885; doi:10.3390/cells11091527)
Supplement: Supplementary file 1 [file cells-11-01527-s001.zip › Supplementary Figure S3.pptx]

## Slide 1
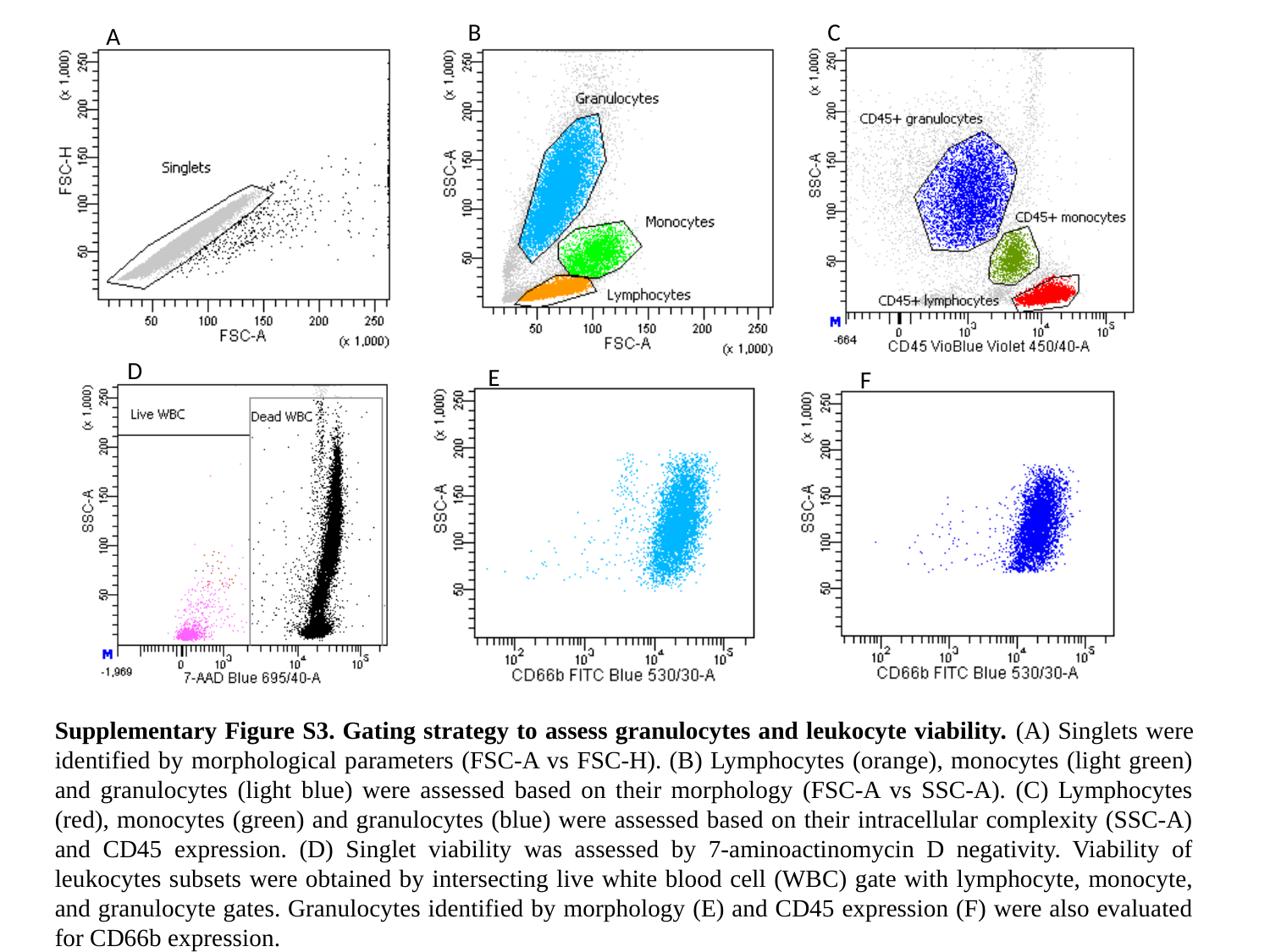

C
B
A
D
E
F
Supplementary Figure S3. Gating strategy to assess granulocytes and leukocyte viability. (A) Singlets were identified by morphological parameters (FSC-A vs FSC-H). (B) Lymphocytes (orange), monocytes (light green) and granulocytes (light blue) were assessed based on their morphology (FSC-A vs SSC-A). (C) Lymphocytes (red), monocytes (green) and granulocytes (blue) were assessed based on their intracellular complexity (SSC-A) and CD45 expression. (D) Singlet viability was assessed by 7-aminoactinomycin D negativity. Viability of leukocytes subsets were obtained by intersecting live white blood cell (WBC) gate with lymphocyte, monocyte, and granulocyte gates. Granulocytes identified by morphology (E) and CD45 expression (F) were also evaluated for CD66b expression.
