# Supplementary material for: Comparison of Whole Blood Cryopreservation Methods for Extensive Flow Cytometry Immunophenotyping
Source: Cells. 2022 May 2;11(9):1527. doi: 10.3390/cells11091527 (PMC9103885; doi:10.3390/cells11091527)
Supplement: Supplementary file 1 [file cells-11-01527-s001.zip › Supplementary Figure S4.pdf]

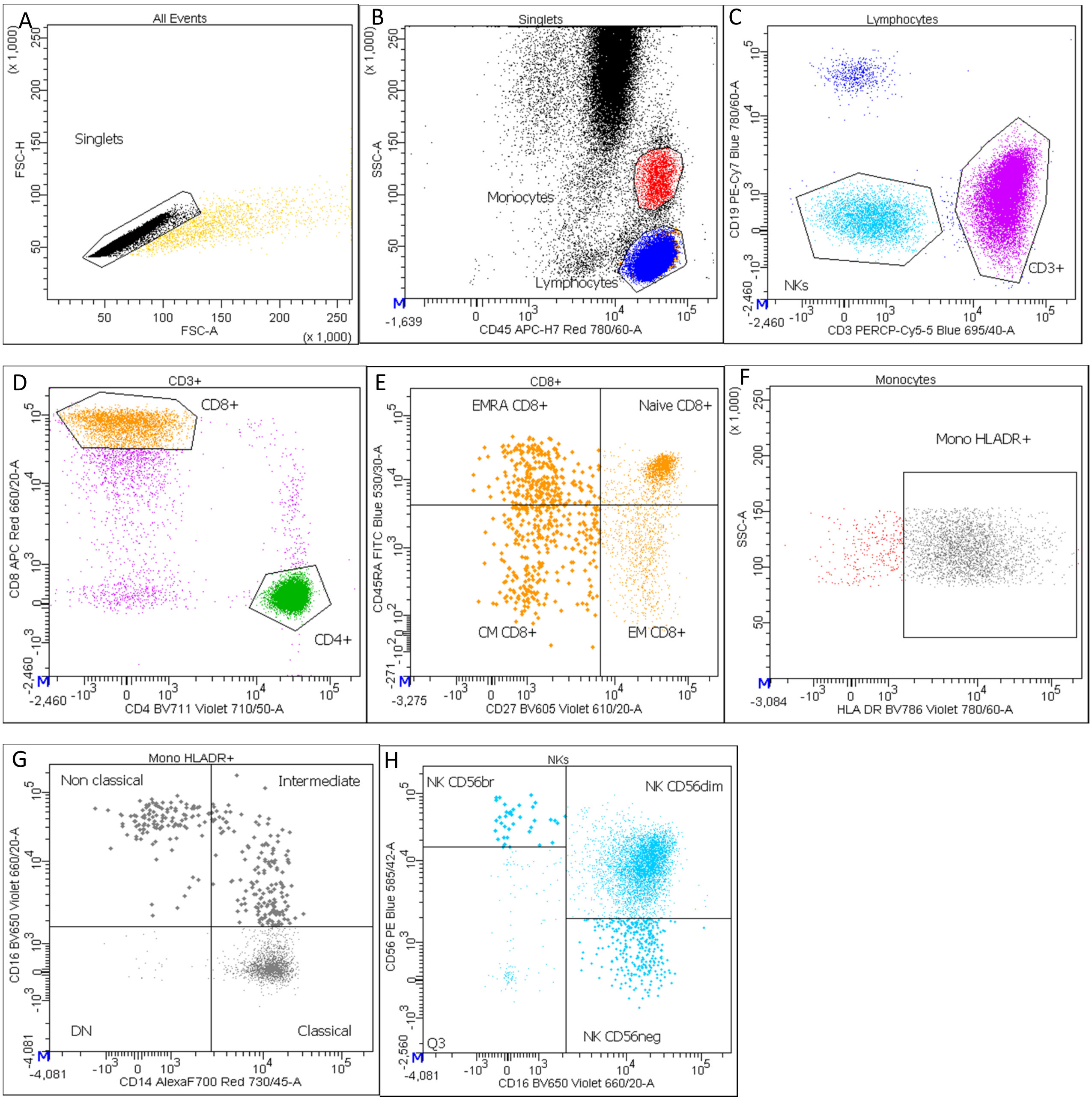

Supplementary Figure S4. M-8-NK gating strategy representative of a fresh processed sample. (A) Single cells (black) were identified by morphological parameters. (B) lymphocytes (blue) and monocytes (red) were assessed based on their morphology and CD45 expression; (C) within lymphocytes, T cells (CD3+ CD19−, violet) and Natural Killers (CD3− CD19−, light blue) were also evaluated; (D) T lymphocytes expressing CD8 antigen (cytotoxic T cells, orange) were identified, then (E) further subdivided based on CD27 and CD45RA expression into naïve (CD45RA+ CD27+), memory cells re-expressing CD45RA (EMRA, CD45RA+ CD27−), Central Memory (CM, CD45RA− CD27−) and Effector Memory (EM, CD45RA− CD27+) subsets; (F) morphologically identified monocytes were assessed for the HLA-DR expression (grey), then divided into (G) classical (CD14+ CD16−), intermediate (CD14+ CD16+) and non- classical (CD14− CD16+) subsets; (H) CD56neg (CD16+ CD56−), CD56dim (CD16+ CD56dim) and CD56bright (CD16− CD56bright) NK subsets were also measured.
